# Successful treatment of an ectopic pregnancy with elevated serum *β*-hCG levels (7,989.00 IU/L) using pure traditional Chinese medicine: a case report

**DOI:** 10.3389/fgwh.2026.1846529

**Published:** 2026-05-29

**Authors:** Jianjun Yu, Chenyi Wu

**Affiliations:** 1 Department of Traditional Chinese Medicine, Guangdong Provincial People's Hospital, Zhuhai Hospital (Jinwan Central Hospital of Zhuhai) Zhuhai, China; 2 Department of Internal Chinese Medicine, Shenzhen Sihui Zhiyun Traditional Chinese Medicine Clinic, Shenzhen, China

**Keywords:** case report, conservative management, ectopic pregnancy, high beta-human chorionic gonadotropin, traditional Chinese medicine treatment

## Abstract

**Background:**

Ectopic pregnancy, particularly tubal pregnancy, is a potentially life-threatening condition that often requires prompt intervention. Although methotrexate (MTX) is commonly used for conservative management, cases with high serum beta-human chorionic gonadotropin (*β*-hCG) levels and visible gestational sac are typically considered beyond the scope of medical therapy and often necessitate surgery. This report describes a patient with a tubal ectopic pregnancy presenting with high serum *β*-hCG levels (7,785.00 IU/L) and a visible gestational sac who was successfully treated with pure Chinese herbal medicine after refusing both surgery and MTX.

**Case presentation:**

A 31-year-old female patient presented with a left fallopian tube ampullary ectopic pregnancy. Her serum *β*-hCG peak reached 7,785.00 IU/L, and a complete gestational sac was visible on ultrasound, exceeding conventional guidelines for medical conservative management. After the patient explicitly refused surgery and methotrexate therapy, a pure Chinese herbal compound treatment was administered, employing the principles of warming yang, invigorating qi, resolving stasis, and eliminating masses. Following treatment, the patient's serum *β*-hCG level rapidly decreased by 32% within 48 h and dropped to 55.07 IU/L by day 14. During treatment, endometrial tissue was expelled. Post-treatment, the ectopic pregnancy mass was completely absorbed, menstruation resumed on schedule, and the associated ovarian cyst resolved.

**Conclusion:**

This case demonstrates the feasibility of pure Chinese herbal medicine for treating ectopic pregnancies with high serum *β*-hCG levels and a visible gestational sac — features that would conventionally be considered unsuitable for medical conservative management. It also highlights the potential advantages of traditional Chinese medicine in holistically regulating the pelvic environment and promoting tissue resorption.

## Introduction

Ectopic pregnancy is one of the leading causes of maternal mortality during early gestation. Currently, serum beta-human chorionic gonadotropin (*β*-hCG) levels serve as the core laboratory indicator guiding treatment selection. Studies indicate that elevated initial *β*-hCG levels are a primary predictor of failure in single-dose methotrexate (MTX) therapy (1). When *β*-hCG exceeds 5,000 IU/L, the failure rate of MTX drug therapy increases significantly, and surgical intervention is typically recommended as the preferred option (2). Traditional Chinese medicine is often used as an adjunctive therapy in ectopic pregnancy treatment, combined with MTX to mitigate side effects and promote mass resorption. However, cases of successful Chinese herbal medicine as monotherapy for ectopic pregnancies with high *β*-hCG levels (especially >5,000 IU/L) and intact gestational sacs, supported by detailed serological and imaging follow-up evidence, are still extremely rare in international literature. This report aims to detail the complete process of such a successful treatment and explore its clinical significance.

## Case presentation

### Patient information and chief complaint

A 31-year-old Asian woman, gravida 2, para 1, presented with 47 days of amenorrhea and mild left lower abdominal pain.

### Medical history

**Figure 1:**
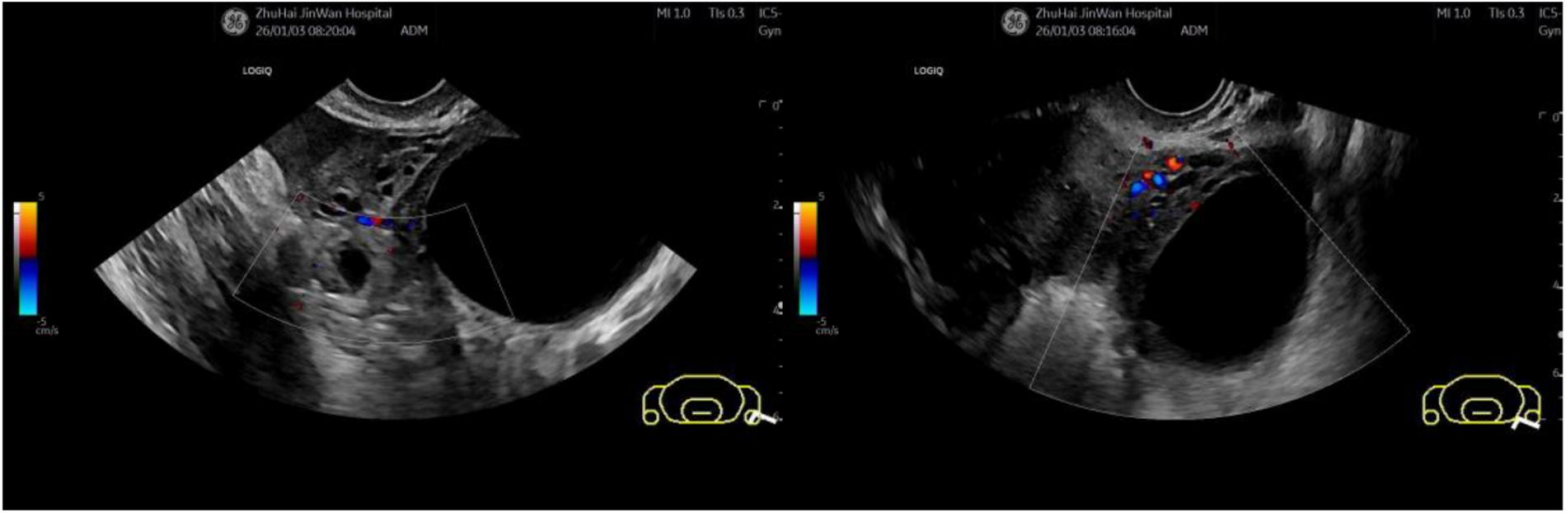
Pre-treatment transvaginal ultrasound (January 3, 2026). A 21 × 15 mm mixed echogenic mass adjacent to the left ovary contains a 10 × 6 mm gestational sac with a yolk sac and peripheral blood flow signals.

She had regular 30-day menstrual cycles; her last menstrual period was November 16, 2025. A home pregnancy test was positive. On January 1, 2026, serum *β*-hCG was 7,989.00 IU/L. On January 3, she developed paroxysmal left lower quadrant pain without vaginal bleeding, nausea, or vomiting. Repeat *β*-hCG was 7,785.00 IU/L. Transvaginal ultrasound revealed no intrauterine gestational sac; a 21 × 15 mm mixed echogenic mass was present in the left adnexa, containing a 10 × 6 mm gestational sac with a yolk sac (Figure 1). No embryonic pole or cardiac activity was detected. The endometrium was thickened (23 mm) with heterogeneous echogenicity, and a 52 × 36 mm anechoic cyst was noted in the left ovary. The patient was admitted with a diagnosis of ectopic pregnancy. Her past medical history was unremarkable, with no history of pelvic inflammatory disease or prior surgeries.

### Clinical examinations

Vital signs were stable. Gynecological examination revealed normal external genitalia, vagina, and cervix; no cervical motion tenderness; mild left adnexal tenderness without palpable mass. Laboratory tests on admission: *β*-hCG 7,785.00 IU/L, progesterone 18.36 ng/mL.

### Diagnosis

Left fallopian tube ampullary ectopic pregnancy.

### Treatment decision

Given that the patient's serum *β*-hCG level (7,785.00 IU/L) significantly exceeded the upper limit for medical conservative management (typically 5,000 IU/L for MTX therapy) (3), and the presence of a complete gestational sac on ultrasound, surgical intervention was clinically recommended (3). The patient explicitly refused both surgery and methotrexate therapy but requested a trial of pure Chinese herbal medicine. Informed consent was obtained.

### Safety protocol

The patient received treatment as an inpatient at Guangdong Provincial People's Hospital, Zhuhai Hospital, a tertiary hospital with a fully equipped gynecology emergency unit and 24-hour surgical backup. Vital signs (blood pressure, heart rate, respiratory rate, temperature) were monitored every 8 h during the active treatment phase (days 0–14). Serial serum *β*-hCG levels were measured every other day for the first 10 days, and then weekly thereafter. Transvaginal ultrasounds were performed on day 0, day 5, day 14, and at final follow-up to assess the ectopic mass and to detect any free fluid in the pouch of Douglas.

The following emergency conversion criteria were predefined before treatment initiation: Acute severe abdominal pain; Hemodynamic instability (systolic blood pressure <90 mmHg or heart rate >110 bpm, or orthostatic dizziness); Ultrasound evidence of moderate-to-large amounts of free fluid in the peritoneal cavity; A sudden rise or persistent plateau in serum *β*-hCG after an initial decline; Patient request for surgical intervention at any time.

A gynecology emergency surgical team was on standby 24/7 throughout the treatment period. The patient was counseled daily about warning symptoms (sudden sharp abdominal pain, shoulder tip pain, syncope, heavy vaginal bleeding) and instructed to report any such symptoms immediately. Throughout the 14-day treatment course, none of these emergency criteria were met, and no signs of tubal rupture occurred.

### Traditional Chinese medicine intervention

TCM examination revealed a pale tongue, thin white coating, a slippery pulse, and a history of cold intolerance. The syndrome was diagnosed as “yang deficiency with blood stasis, mass accumulation internally retained.” The treatment principle was to warm yang, tonify qi, activate blood, resolve stasis, and eliminate the mass. A modified combination of Jiawei Shaofu Zhuyu Decoction and Ectopic Pregnancy Formula II was prescribed. The full composition is detailed in Table 1. The herbal formula was prescribed in packets. One packet was decocted into two oral doses. The dosing frequency was dynamically adjusted: one packet per day (two doses) for days 1–4; one packet every other day (one dose per day) for days 5–10; and one packet every 4 days (one dose every two days) for days 11–14. Total treatment duration was 14 days. The background and original compositions of the base formulae are provided in Appendix 1.

**Table 1:** Composition of the Chinese herbal formula.

Chinese name	English name	Botanical origin	Content (g)	Special instructions
生黄芪	Astragali radix	*Astragalus membranaceus* (Fisch.) Bge. var. *mongholicus* (Bge.) Hsiao	100	-
炮附片	Aconiti Radix Lateralis Praeparata	*Aconitum carmichaelii* Debx.	25	decocted first
天花粉	Trichosanthis Radix	*Trichosanthes kirilowii* Maxim.	20	-
柴胡	Bupleuri Radix	*Bupleurum chinense* DC.	15	-
当归	Angelicae Sinensis Radix	*Angelica sinensis* (Oliv.) Diels	10	-
桃仁	Persicae Semen	*Prunus persica* (L.) Batsch	12	-
红花	Carthami Flos	*Carthamus tinctorius* L.	12	-
川芎	Chuanxiong Rhizoma	*Ligusticum chuanxiong* Hort.	18	-
赤芍	Paeoniae RadixRubra	*Paeonia lactiflora* Pall.	15	-
生五灵脂	Faeces Trogopterori	*Trogopterus xanthipes*	20	wrapped in bag for decoction
三棱	Sparganii Rhizoma	*Sparganium stoloniferum* Buch.-Ham.	15	-
莪术	Curcumae Rhizoma	*Curcuma phaeocaulis* Val.	15	-
水蛭	Hirudo	*Whitmania pigra* Whitman	6	-
蜈蚣	Scolopendra	*Scolopendra subspinipes mutilans* L. Koch	5	-
川牛膝	Cyathulae Radix	*Cyathula officinalis* Kuan	15	-
肉桂	Cinnamomi Cortex	*Cinnamomum cassia* Presl	10	added later
干姜	Zingiberis Rhizoma	*Zingiber officinale* Rosc.	12	-
制吴茱萸	Evodiae Fructus	*Euodia rutaecarpa* (Juss.) Benth	15	-
盐小茴香	Foeniculi Fructus	*Foeniculum vulgare* Mill.	6	-
桂枝	Cinnamomi Ramulus	*Cinnamomum cassia* Presl	15	-
人参	Ginseng Radix et Rhizoma	*Panax ginseng* C. A. Mey.	15	decocted first
茯苓	Poria	*Poria cocos* (Schw.) Wolf	15	-
败酱草	Herba Patriniae	*Patrinia scabiosifolia* Fisch. ex Trevir.	20	-
大黄	Rhei Radix et Rhizoma	*Rheum palmatum* L	15	-
炙甘草	Glycyrrhizae Radix et Rhizoma Praeparata cum Melle	*Glycyrrhiza uralensis* Fisch	12	-
丹参	Salviae Miltiorrhizae Radix et Rhizoma	*Sal*via *miltiorrhiza* Bge.	15	-

### Treatment response and follow-up

Serological response (as shown in Table 2): *β*-hCG fell dramatically from 7,785.00 IU/L (day 0) to 5,289.00 IU/L on day 3, a 32% decrease within 48 h, indicating a rapid therapeutic response. By day 5, it had dropped to 1,349.62 IU/L (an 83% decrease from baseline), falling below the conventional safe threshold. Finally, on day 14, *β*-hCG reached 55.07 IU/L, approaching non-pregnant levels.

**Table 2:** Dynamic changes in serum *β*-hCG levels during treatment.

Date	Treatment days	Serum *β*-hCG (IU/L)
2026-01-01	Pre-treatment	7,989.00
2026-01-03	Treatment Start Date (Day 0)	7,785.00
2026-01-05	Day 3	5,289.00
2026-01-07	Day 5	1,349.62
2026-01-09	Day 7	595.20
2026-01-17	Day 14	55.07
2026-02-06	Clinically cured	No follow-up

Clinical symptoms: Between days 7 and 12, the patient passed a membranous, sac-like tissue (consistent with necrotic endometrium) accompanied by mild vaginal bleeding, which later resolved. No further abdominal pain or signs of intra-abdominal hemorrhage occurred. The patient tolerated the herbal decoction well throughout the 14-day treatment period. She reported no adverse effects, including nausea, vomiting, diarrhea, abdominal bloating, headache, dizziness, or skin rash. The only minor complaint was the bitter taste of the medicine, which did not affect her compliance.

**Figure 2:**
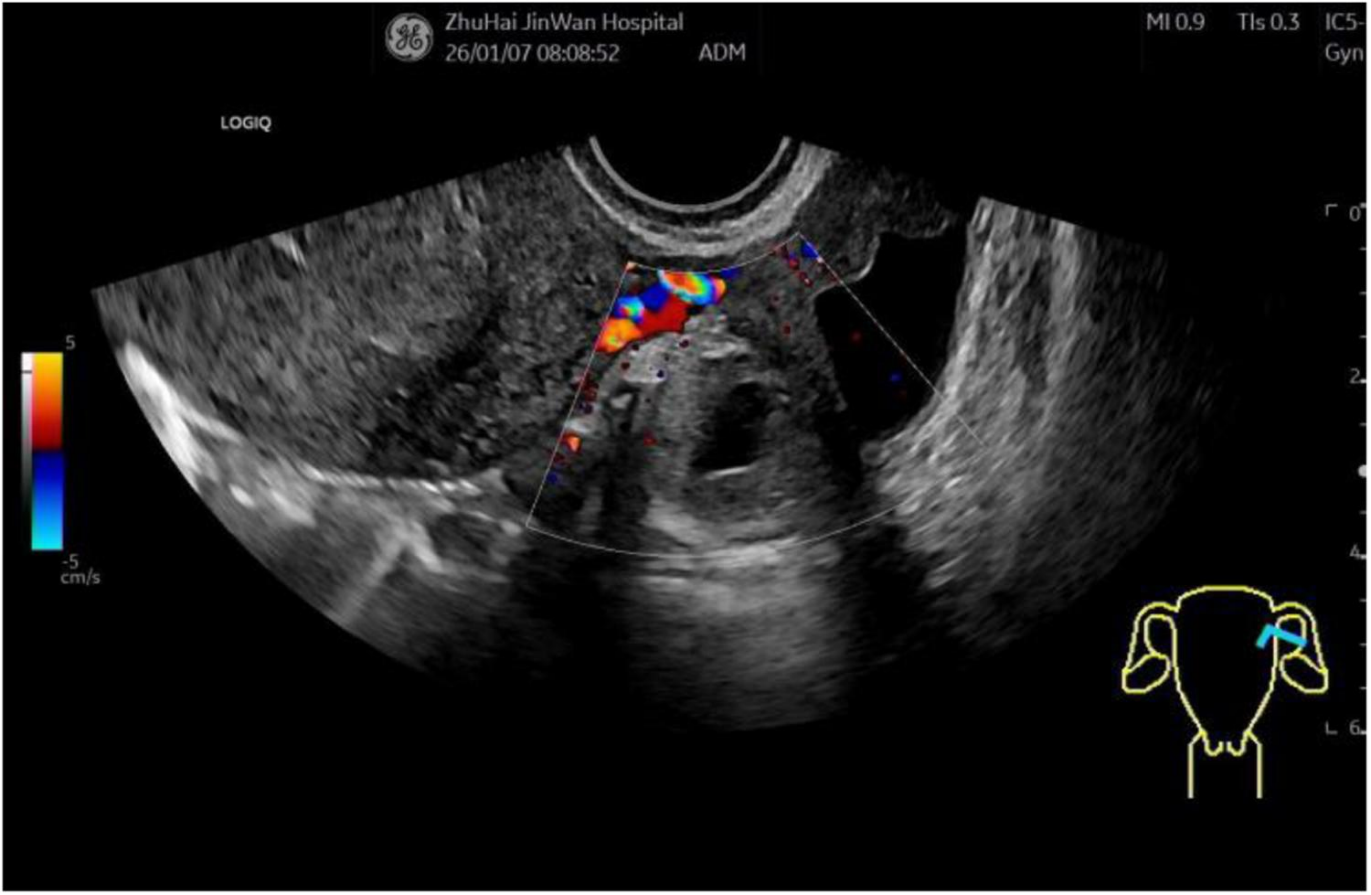
Transvaginal ultrasound on day 5 of treatment (January 7, 2026). The ectopic mass transiently enlarged to 24 × 20 mm with an irregular gestational sac, suggesting necrosis and hemorrhage.

**Figure 3:**
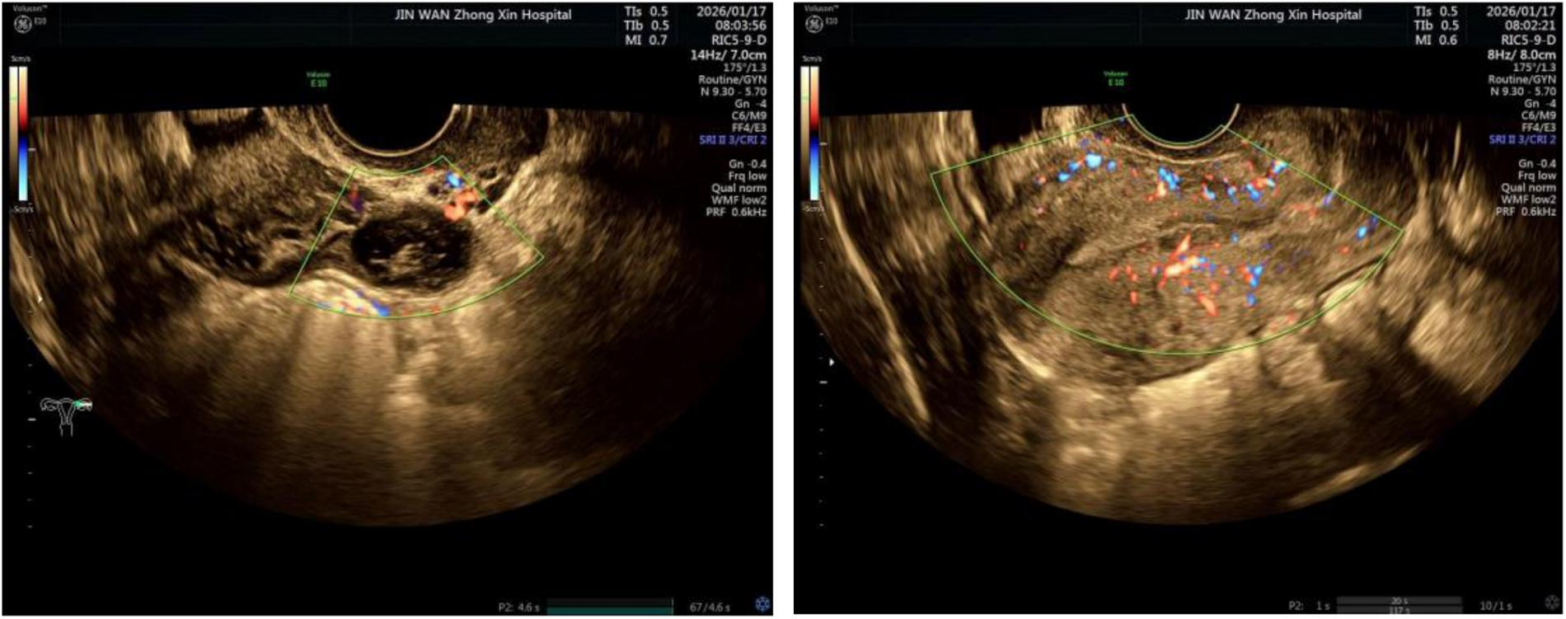
Transvaginal ultrasound on day 14 of treatment (January 17, 2026). The ectopic mass decreased to 20 × 18 mm with peripheral blood flow signals reduced to punctate and linear patterns, indicating the absorption phase.

Imaging evolution: On day 5, the ectopic mass transiently enlarged to 24 × 20 mm with an irregular gestational sac, suggesting necrosis and hemorrhage (Figure 2). By day 14, the mass had shrunk to 20 × 18 mm with reduced blood flow signals (Figure 3). Endometrial thickness normalized to 10 mm, and the left ovarian cyst resolved completely by February 6. All serial ultrasounds showed no free fluid in the pouch of Douglas, confirming absence of rupture.

**Figure 4:**
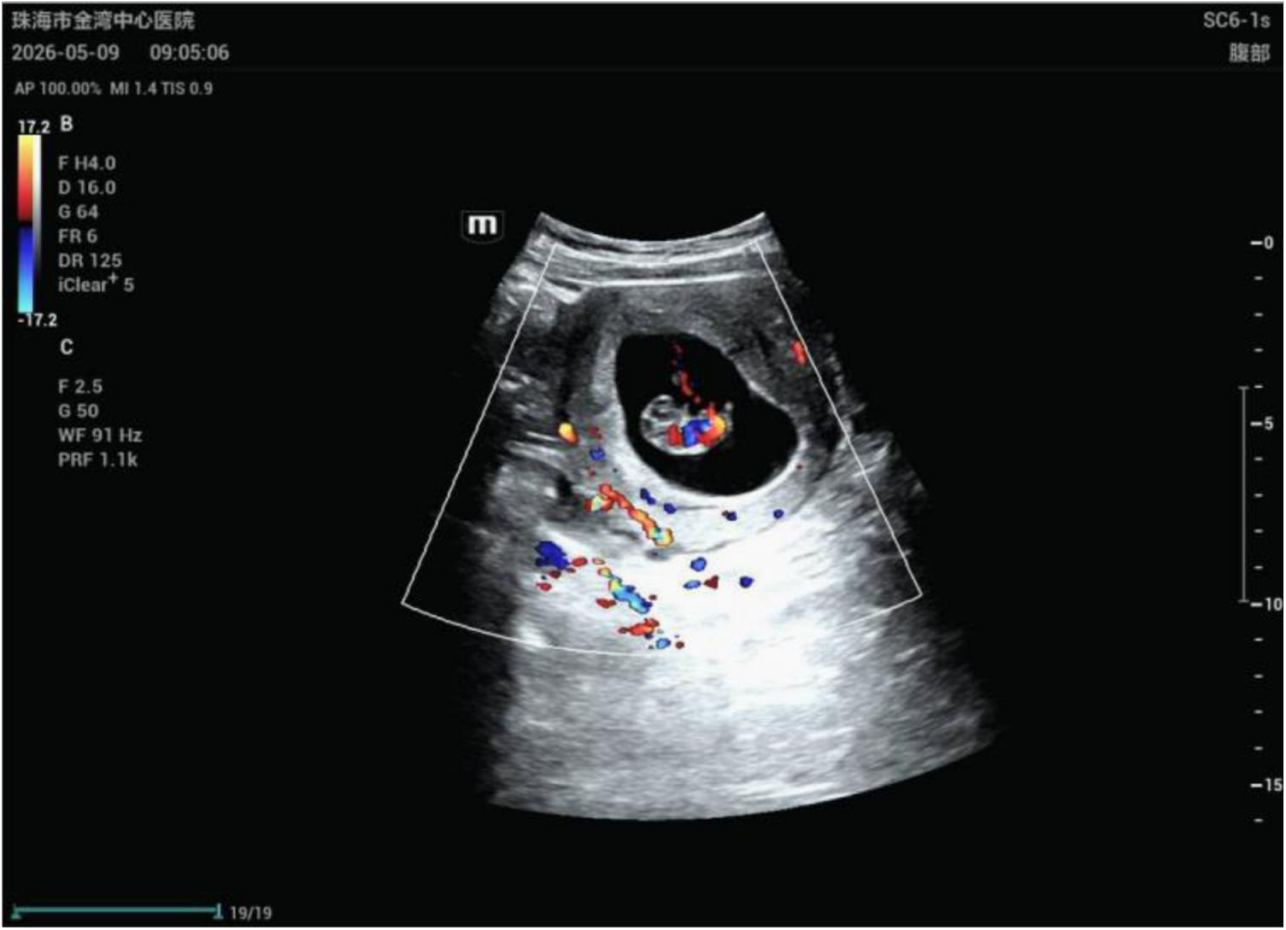
Transvaginal ultrasound on May 9, 2026. A viable intrauterine pregnancy at approximately 9 + weeks of gestation shows a gestational sac (57 × 41 mm), embryo (crown-rump length 25 mm), and regular fetal heartbeat.

Long-term outcome: Menstruation resumed on schedule on February 3, 2026. Her next menstrual cycle occurred on March 5, 2026, and she subsequently conceived naturally. On May 9, 2026, transvaginal ultrasound confirmed a viable intrauterine pregnancy at approximately 9 + weeks of gestation, showing a gestational sac measuring 57 × 41 mm with a yolk sac and an embryo (crown-rump length 25 mm). A regular fetal heartbeat was detected on color Doppler. Both ovaries were normal in size and morphology, and no adnexal masses were identified (Figure 4). These findings definitively confirm complete resolution of the prior ectopic pregnancy and exclude any persistent trophoblastic activity.

## Discussion

This case provides a critical exception to the notion that extremely high *β*-hCG levels in ectopic pregnancy necessitate surgery. The patient's *β*-hCG peaked at 7,989 IU/L, and ultrasound revealed a complete gestational sac with a yolk sac—features typically considered contraindications to conservative management. Nevertheless, pure Chinese herbal medicine achieved rapid and successful resolution. Although pre-treatment *β*-hCG had declined spontaneously from its peak to 7,785 IU/L (with a concurrent drop in progesterone), the level remained far above the conventional safety threshold. Thus, for carefully selected patients who decline standard therapy, pure Chinese herbal medicine may serve as an effective and safe exploratory option for managing ectopic pregnancies with markedly elevated *β*-hCG levels and sonographic evidence of a gestational sac.

The herbal regimen initiated an orderly pathological reversal process. A rapid biochemical response occurred within 48 h: *β*-hCG decreased by 32% from baseline, a decline rate comparable to that of effective methotrexate therapy (1, 2), suggesting direct or indirect suppression of trophoblast activity. This was followed by characteristic clinical and imaging changes: concurrent with the dramatic *β*-hCG drop, ultrasound showed transient enlargement of the ectopic mass, likely reflecting internal hemorrhage, necrosis, and inflammatory edema—heralding subsequent resorption. The patient then expelled decidual tissue vaginally, a classic sign of halted embryonic activity and hormonal withdrawal, directly demonstrating the “activating blood and resolving stasis” effects of TCM. Ultimately, the ectopic mass was completely absorbed, the associated ovarian cyst resolved, the thickened endometrium normalized, and menstruation resumed. This complete chain—from biochemical changes to tissue repair and functional restoration—shows that the treatment not only eliminated the lesion but also promoted holistic pelvic recovery.

The success of this case relied on precise differentiation of a complex pathogenesis. Rather than simplistically equating ectopic pregnancy with “blood stasis,” we identified a unique pattern of “yang deficiency with blood stasis.” The patient's pale tongue, habitual cold intolerance, cold limbs, and slightly weak cun pulse indicated underlying kidney yang deficiency and lower jiao deficiency with cold. This aligns with the TCM understanding that ectopic pregnancy often involves composite pathogenesis of “kidney deficiency with blood stasis” or “yang deficiency with blood stasis” (4). Thus, yang deficiency (impaired warming and transporting) constitutes the root cause, while blood stasis accumulation is the branch manifestation. Recognizing this “root deficiency” as the foundation for “branch excess” guided the correct therapeutic approach.

Consequently, treatment did not rely on potent stasis-attacking herbs alone (which could further damage yang qi). Instead, we established the principle of “warming yang and tonifying qi to address the root, while activating blood and resolving masses to address the branch.” This “fortify the healthy qi and dispel pathogenic factors” strategy aligns with TCM principles for gynecological masses (5). The formula contained a high dose (100 g) of raw Astragali radix, together with Ginseng Radix, Aconiti Radix Lateralis, and Cinnamomi Cortex, to vigorously warm kidney yang, tonify primordial qi, and enhance pelvic qi and blood circulation—providing the energetic basis for stasis resolution while securing the root. On this foundation, we added Sparganii Rhizoma, Curcumae Rhizoma, Hirudo, Scolopendra, and Trichosanthis Radix, which break blood stasis, eliminate masses, and act directly on embryonic tissue, allowing precise targeting without damaging vital qi. Modern research confirms that trichosanthin (the active component of Trichosanthis Radix) specifically induces apoptosis in trophoblast cells (6, 7), and that Hirudo and Curcumae Rhizoma possess anticoagulant and anti-angiogenic effects (8, 9), which may help disrupt blood supply to the ectopic embryo and promote mass absorption.

While trichosanthin-induced trophoblast apoptosis provides a direct anti-embryonic effect, the successful resolution of this high-risk ectopic pregnancy cannot be attributed to a single mechanism. The herbal formula's design embodies a sophisticated “multi-target, multi-pathway” strategy that addresses both the pathological mass (branch manifestation) and the patient's underlying constitutional weakness (root cause).

The inclusion of 100 g of raw Astragali Radix serves a dual purpose. From a TCM perspective, Astragalus tonifies Spleen Qi to achieve hemostasis and reinforces Defensive Qi to contain the inflammatory response at the ectopic site (10, 11). Modern pharmacological studies have demonstrated that Astragalus polysaccharides exhibit immunomodulatory and anti-inflammatory effects, which may underlie its traditional use in reinforcing the body's defensive capacity (12). The patient's uneventful clinical course—only mild vaginal bleeding without any signs of hemodynamic instability or intra-abdominal hemorrhage—supports this theoretical framework.

The combination of Hirudo and Curcumae Rhizoma provides potent anti-angiogenic and anti-thrombotic effects. Pharmacological studies have demonstrated that Hirudo extracts inhibit platelet aggregation (8), while Curcumae Rhizoma exhibits anti-angiogenic properties that may help disrupt the blood supply to the ectopic trophoblastic tissue (9).

The clinical significance of this “Yiqi Huoxue” strategy is supported by recent studies. Jia et al. (13) reported that adding a Huoxue Huayu Decoction to MTX significantly shortened the time for ectopic mass absorption (40.26 ± 12.35 days vs. 48.79 ± 15.36 days, *P* < 0.05) and improved tubal patency rates (52.00% vs. 29.27%, *P* < 0.05) compared to MTX alone. Similarly, Wang et al. (14) demonstrated that combining Ectopic Pregnancy Formula No. 2 with intratubal MTX accelerated gestational sac resorption (1.2 ± 0.7 months vs. 3.6 ± 1.7 months, *P* < 0.01) and reduced tubal obstruction rates (10.5% vs. 43.8%, *P* < 0.05). These findings corroborate our observation that the addition of Chinese herbal medicine not only accelerates biochemical resolution but also promotes structural regression of the ectopic mass and preserves tubal function.

Consistent with the principle of “withdrawing therapy upon substantial resolution” from the Suwen (15), we dynamically adjusted the dosage based on *β*-hCG decline.

During the initial phase (days 1–4), medication was given twice daily to concentrate therapeutic effect. Once a sharp *β*-hCG decline was confirmed (day 5), dosing was reduced to once daily, shifting focus to consolidating the root and avoiding excessive attack. In the later phase (days 11–14), the dose was further reduced to once every two days to support mass resorption and pelvic repair. This real-time adjustment based on objective laboratory feedback integrates modern monitoring technology with traditional Chinese medical wisdom, embodying precision and safety in personalized treatment.

This case presents a novel approach of pure herbal medicine under strict monitoring for patients with contraindications to surgery, those who refuse standard therapy, or those who have failed MTX treatment. It demonstrates the potential of personalized TCM regimens based on precise pattern differentiation for acute gynecological conditions. The main limitation is its single-case nature; findings should not be generalized without caution. The complex mechanisms of action require further pharmacological research to identify key target molecules and pathways. Although serum *β*-hCG was not followed until <5 IU/L, clinical cure was definitively confirmed by complete mass resolution, return of normal menstruation, and subsequent successful intrauterine pregnancy. These outcomes provide compelling evidence of complete resolution without persistent trophoblastic activity.

## Conclusion

This case demonstrates that, provided rigorous monitoring is implemented and the practitioner has extensive experience in managing acute gynecological emergencies with TCM, a pure herbal treatment approach based on warming yang, tonifying qi, resolving stasis, and eliminating masses may serve as an effective conservative strategy for carefully selected ectopic pregnancy patients with elevated *β*-hCG levels. This approach achieves biochemical reversal, lesion resorption, and preservation of reproductive function. These findings serve as an important reference for expanding clinical treatment options for ectopic pregnancy.

## Patient perspective

The patient stated that when she first learned she had an ectopic pregnancy with such a high *β*-hCG level, she was very frightened, especially after the doctor explained that surgery would usually be necessary. She was grateful that the doctor respected her wish to try traditional Chinese medicine instead. She felt reassured by the doctor's patient explanation and the careful monitoring throughout the treatment. Although she experienced some vaginal bleeding during the treatment, she was relieved that the bleeding stopped on its own and that she never needed surgery. She was deeply grateful to the medical team for helping her recover completely and for preserving her fertility. She now has returned to normal menstruation and feels healthy again.

## Data Availability

The original contributions presented in the study are included in the article/Supplementary Material, further inquiries can be directed to the corresponding author.

## Appendix 1. Background and Rationale of the Herbal Formula

**Source of the base formulae**

Shaofu Zhuyu Decoction (少腹逐瘀汤) is a classical formula first recorded in Yi Lin Gai Cuo (Corrections on the Errors of Medical Works, 1830) by Wang Qingren, a renowned physician of the Qing Dynasty. It is traditionally indicated for blood stasis with cold in the lower abdomen. The original composition is as follows:

**Table:**

Chinese Name	English name	Botanical origin	Original Dose	Conversion (g)	Special Instructions
小茴香	Foeniculi Fructus	*Foeniculum vulgare* Mill.	7 pieces	1.5 g	stir-fried
干姜	Zingiberis Rhizoma	Zingiber officinale Rosc.	2 fen	0.6 g	stir-fried
延胡索	Corydalis Rhizoma	*Corydalis yanhusuo* W. T. Wang	1 qian	3 g	-
没药	Myrrha	*Commiphora myrrha* Engl.	1 qian	3 g	-
当归	Angelicae Sinensis Radix	*Angelica sinensis* (Oliv.) Diels	3 qian	9 g	-
川芎	Chuanxiong Rhizoma	*Ligusticum chuanxiong* Hort.	1 qian	3 g	-
官桂	Cinnamomi Cortex	*Cinnamomum cassia* Presl	1 qian	3 g	-
赤芍	Paeoniae RadixRubra	*Paeonia lactiflora* Pall.	2 qian	6 g	-
蒲黄	Typhae Pollen	*Typha angustifolia* L.	3 qian	9g	raw
五灵脂	Faeces Trogopterori	*Trogopterus xanthipes*	2 qian	6 g	stir-fried

Source: Wang QR Yi Lin Gai Cuo. Beijing: People's Medical Publishing House; 2005. (Original work published 1830)

Ectopic Pregnancy Formula II (宫外孕Ⅱ号方) is a modern TCM formula developed by the First Affiliated Hospital of Shanxi Medical College in the 1970s for the conservative treatment of ectopic pregnancy. The original composition is as follows:

**Table:**

Chinese Name	English name	Botanical origin	Content (g)	Special Instructions
丹参	Salviae Miltiorrhizae Radix et Rhizoma	*Sal*via *miltiorrhiza* Bge.	15 g	stir-fried
赤芍	Paeoniae Radix Rubra	*Paeonia lactiflora Pall.*	15 g	stir-fried
桃仁	Persicae Semen	*Prunus persica* (L.) Batsch	9 g	-
三棱	Sparganii Rhizoma	*Sparganium stoloniferum* Buch.-Ham.	1.5–6 g	-
莪术	Curcumae Rhizoma	*Curcuma phaeocaulis* Val	1.5–6 g	-

Source: Shanxi Medical College Affiliated First Hospital. Conservative treatment of ectopic pregnancy with integrated traditional Chinese and Western medicine. Chin J Obstet Gynecol. 1975;10(3):215–218.

**Rationale for modifications**

High-dose Astragali radix (100 g) was added to strongly tonify qi and stabilize the body's vital energy, preventing excessive attack from the blood-breaking herbs.

Hirudo and Scolopendra were added to enhance the effect of “breaking blood and eliminating mass,” based on the severity of the ectopic mass and the high *β*-hCG level.

Warming herbs (Aconiti lateralis radix praeparata, Cinnamomi cortex, Zingiberis rhizoma, Evodiae fructus) were emphasized to address the patient's yang deficiency constitution.

Conversely, certain herbs commonly found in the base formulae were deliberately omitted:

Myrrha, Corydalis Rhizoma, and Typhae Pollen — key components of Shaofu Zhuyu Decoction — were not included. These herbs are primarily used for promoting qi circulation and relieving pain. Given that the patient's abdominal pain was mild and not the dominant clinical issue, the therapeutic strategy prioritized warming yang and strongly resolving blood stasis over simple qi regulation and analgesia. The omission reflects a shift in therapeutic focus from “pain relief” to “root cause elimination.”
